# Sanger Sequencing Reveals Novel Variants in *GLO-1*, *ACE*, and *CBR1* Genes in Patients of Early and Severe Diabetic Nephropathy

**DOI:** 10.3390/medicina60091540

**Published:** 2024-09-20

**Authors:** Syed Zubair Hussain Shah, Amir Rashid, Asifa Majeed, Tariq Ghafoor, Nadeem Azam

**Affiliations:** 1 Department of Biochemistry and Molecular Biology, Army Medical College, National University of Medical Sciences, Rawalpindi 46000, Pakistan; amir.rashid@numsak.edu.pk (A.R.); asifa_pak@yahoo.com (A.M.); 2 Armed Forces Bone Marrow Transplant Center, Rawalpindi 46000, Pakistan; drtariqghafoor@gmail.com; 3 Pak Emirates Military Hospital, Rawalpindi 46000, Pakistan

**Keywords:** diabetic nephropathy, genetic variations, *GLO1*, *ACE*, *CBR1*

## Abstract

*Background and Objectives:* Diabetes is a global health issue, with approximately 50% of patients developing diabetic nephropathy (DN) and 25% experiencing early and severe forms of the disease. The genetic factors contributing to rapid disease progression in a subset of these patients are unclear. This study investigates genetic variations in the *GLO-1*, *CBR-1*, and *ACE* genes associated with early and severe DN. *Materials and Methods:* Sanger DNA sequencing of the exons of *CBR1*, *GLO1*, and *ACE* genes was conducted in 113 patients with early and severe DN (defined as occurring within 10 years of the diagnosis of diabetes and with eGFR < 45 mL/min/1.73 m^2^) and 100 controls. The impact of identified genetic variations was analyzed using computational protein models created in silico with SWISS-Model and SWISS-Dock for ligand binding interactions. *Results:* In *GLO1*, two heterozygous missense mutations, c.102G>T and c.147C>G, and one heterozygous nonsense mutation, c.148G>T, were identified in patients. The SNP rs1049346 (G>A) at location 6:38703061 (GRCh38) was clinically significant. The c.147C>G mutation (C19S) was associated with ligand binding disruption in the GLO1 protein, while the nonsense mutation resulted in a truncated, non-functional protein. In *CBR1*, two heterozygous variations, one missense c.358G>A, and one silent mutation c.311G>C were observed, with the former (D120N) affecting the active site. No significant changes were noted in *ACE* gene variants concerning protein structure or function. *Conclusions:* The study identifies four novel and five recurrent mutations/polymorphisms in *GLO1*, *ACE*, and *CBR1* genes associated with severe DN in Pakistani patients. Notably, a nonsense mutation in *GLO1* led to a truncated, non-functional protein, while missense mutations in *GLO1* and *CBR1* potentially disrupt enzyme function, possibly accelerating DN progression.

## 1. Introduction

Diabetes mellitus is a major global healthcare challenge and one of the fastest-growing diseases worldwide. In 2021, an estimated 536.6 million people (10.5% of the global population) were living with diabetes, a number projected to rise to 783.2 million (12.2%) by 2045, according to the International Diabetes Federation (IDF) [1]. Diabetes mellitus can result in a serious complication, diabetic nephropathy (DN), which manifests in approximately 50% of patients with type 2 diabetes mellitus [2]. There are multiple factors implicated in the development of diabetes and its complications, i.e., poor diet, genetic makeup, environmental factors, an unhealthy lifestyle, and lack of exercise [3,4]. The underlying biochemical basis has been shortlisted to insulin resistance, chronic low-grade inflammation, oxidative stress, and genetic mutations or variations [3,5]. Complications resulting from diabetes, including DN, are among the leading causes of morbidity and mortality in the affected population [6].

DN is characterized by a reduction in the glomerular filtration rate (GFR) or an increase in albuminuria, and it can progress to end-stage renal disease (ESRD), necessitating hemodialysis or kidney transplantation [7]. Even with treatment, such as with angiotensin receptor blockers, almost half of the patients with diabetic nephropathy progress from microalbuminuria and a maintained GFR to advanced renal failure and frank proteinuria [8]. The mechanisms behind this progression are unclear; however, one implicated factor is the production of advanced glycation end products (AGEs) [9] triggered by methylglyoxal (MG) [10]. MG is an unwanted byproduct of glycolysis being produced normally and needs to be metabolized to relatively inert substances or it can lead to the formation of AGEs. AGEs result from the non-enzymatic covalent binding of sugars to DNA, proteins, or lipids, which are produced physiologically, leading to aging and pathologically leading to diseases such as diabetes [11]. Reactive oxygen species (ROS) cause protein carbonylation, as a major mechanism of ROS damage [12]. Reactive carbonyl species (RCS) are produced when lipids, amino acids, and carbohydrates are continuously oxidized by free oxygen radicals produced normally as a result of metabolism [13]. RCS, including MG, are byproducts of the oxidation of lipids, amino acids, and carbohydrates and are associated with various chronic conditions, such as diabetes and its complications, atherosclerosis, neurodegenerative diseases, and inflammatory disorders [13]. Normally, MG is detoxified by protective enzymes such as glyoxalase-1 (GLO1) and glyoxalase-2 (GLO2) [14]. Previous research has indicated significantly lower levels of glyoxalase-1 in DN patients who experienced accelerated disease progression [15]. However, the SNP rs4746 in the *GLO1* gene in the same patients was not associated with this rapid progression [15]. Given the protective role of GLO1, our study aimed to investigate genetic variations in the *GLO1* gene that might influence enzyme function and contribute to the early onset of DN.

The *CBR1* gene, located on chromosome 21q22.13, codes for the carbonyl reductase-1 enzyme (E.C.1.1.1.184, CBR1), which detoxifies carbonyl compounds including methylglyoxal (MG) [16]. Genetic variations in the *CBR1* gene may modulate the activity of this enzyme and influence susceptibility to DN. Emerging studies have suggested that *CBR1* gene polymorphism is linked with decreased enzyme activity, increased MG, and other carbonyl species, underscoring their potential role in DN pathogenesis [13].

Recent genome-wide association studies (GWAS) have identified genetic variations in the angiotensin-converting enzyme (*ACE)* gene as significant contributors to the pathogenesis of DN, especially insertion/deletion (I/D) polymorphism in intron 16 [17,18]. However, our study employs a more comprehensive approach by examining other segments of the ACE gene using Sanger sequencing, specifically targeting early and severe forms of DN in a different population. Sanger sequencing is chosen for its high accuracy, low cost, long read lengths, and ability to handle low-quality DNA samples, making it the gold standard for detecting single nucleotide variations [19]. The *ACE* gene is involved in renal hemodynamics, blood pressure regulation, and glomerular basement membrane integrity [20]. ACE inhibitors have a proven efficacy in diabetic kidney disease and are the first-line drugs in diabetic nephropathy even in normotensive patients, further highlighting the gene’s role in DN pathogenesis [20,21]. The exact mechanisms by which the *ACE* gene influences the pathogenesis of DN remain unclear [19,22]. Research focusing on ACE gene variations, particularly in populations that are underrepresented in genetic studies, such as the Pakistani population, is essential. Moreover, there is a need to specifically investigate those DN patients who rapidly progress to severe disease, as this subgroup may reveal critical insights into the genetic underpinnings of DN. The exploration of genetic variations in *GLO1*, *CBR1*, and *ACE* genes and their effect on enzyme structure, function, or ligand interaction is imperative in this regard and the main focus of this study.

## 2. Materials and Methods

### 2.1. Patients/Participants

In this case–control study, 113 patients and 100 healthy adults were enrolled. Patients who had diabetes (type 1 or 2) diagnosed for less than 10 years and developed severe renal disease [23] were selected. Both male and female patients between the ages of 18 and 80 years having glycosylated hemoglobin (HbA1c) > 6.5% (as determined by an ELISA) [24] and eGFR < 45 mL/min/1.73 m^2^ [25] were enrolled. Patients who were dependent on hemodialysis within 10 years since diabetes diagnosis were also included [23]. Patients in a coma and those with concomitant illnesses were excluded. The healthy adults participating after providing written informed consent having HbA1c < 6.5% [26], serum urea < 7.1 mmol/L [27], serum creatinine (61.9 to 114.9 µmol/L) for men and (53 to 97.2 µmol/L) for women [27], and eGFR > 60 mL/min/1.73 m^2^ [25] were included in the control group.

### 2.2. Ethics and Compliance

The health of the patients/participants was given foremost priority, and the confidentiality of his/her personal and medical details was ensured. After informed consent was provided in writing, the medical history/records and 10 mL of blood samples for research purposes were obtained. The institutional ethical review committee approved the consent form and information sheet printed in two languages (English and Urdu).

### 2.3. Biochemical Analysis

The concentrations of urea, creatinine, and other biochemical parameters were determined using a fully automatic chemical analyzer (Cobas c311 Productos Roche Inter Americana S.A. (PRISA) Panama) that operates on the principle of the spectrophotometer. The Modification of Diet in Renal Disease (MDRD) equation [28] was used to determine eGFR. 

### 2.4. Genetic Analysis

**Figure 1:**
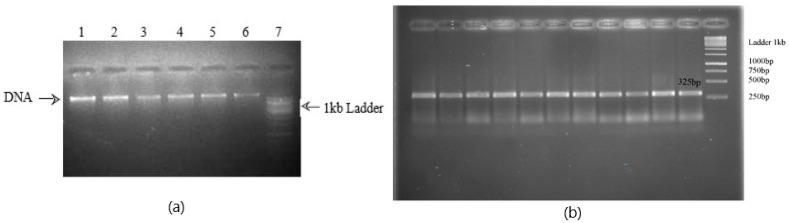
(**a**) A picture of 1% agarose gel showing positive DNA bands on gel electrophoresis. (**b**) A picture of 2% agarose gel showing 325 bp fragments on gel electrophoresis amplified using a PCR.

DNA was extracted using the phenol-chloroform method and kit (FavorPrep Blood Genomic DNA Extraction Mini Kit Cat No. FABGK 001-1 by Favorgen Biotech Corp, Taiwan, China) and checked using 1% agarose gel electrophoresis (Figure 1a). A PCR was carried out after obtaining primers from the Macrogen company (10F,254, Beotkkot-ro, Geumcheon-gu, Seoul, Republic of Korea). The parameters that were targeted while selecting primers online are given in Table 1. The primers used for a few exons are presented in Table 2. The reaction mixture contained reaction buffer at 1X, MgCl_2_ at 0.2 mM, dNTPs at 200 µM, primers at 25 pM each, Taq DNA polymerase at 0.05 units/µL, NF water, and template DNA at approx. 100 ng/µL. These reagents were procured from Thermo Fisher Scientific through NeoTech, an authorized distributor in Lahore, Pakistan. 

The PCR product (50 µL) was checked using 2% agarose gel electrophoresis (Figure 1b) and was purified using the kit (Favor Prep PCR Clean-up Mini Kit Cat. No. FAPCK001) and a sequencing PCR was carried out. Genetic analysis was performed using the Sanger sequencing method on the ABI Genetic Analyzer 3500. 

### 2.5. Statistical Analysis and Software

SPPS version 29.0 was used to analyze the biochemical data. Using Primer 3 Plus software [29], primers for exon sequencing were created. The National Center for Bioinformatics Primer Basic Local Alignment Search Tool (NCBI Primer-BLAST available at https://www.ncbi.nlm.nih.gov/tools/primer-blast/ accessed on 13 February 2024) service was then used to confirm the correctness and single hits of the primers. The chromatograms were interpreted using biological software Finch TV version 1.4 and Bio Edit version 7.2 [30]. 

### 2.6. In Silico Analysis

To look for changes in protein structure and ligand interaction, the computational modeling of identified variations was performed using the online biological software Swiss-Model [31], Swiss Dock [32,33], and Pymol [34]. The amino acid residue conservation in different species over the process of evolution was checked using the conservation alignment tool of the UCSC genome browser available online at (https://genome.ucsc.edu/cgi-bin/hgTracks?db=hg38&lastVirtModeType=default&lastVirtModeExtraState=&virtModeType=default&virtMode=0&nonVirtPosition=&position=chr6%3A38703019%2D38703069&hgsid=2344666432_V7We2em7u7htpAaWN2AHmK74KPkF accessed on 5 September 2024).

## 3. Results

### 3.1. Demographic Profile

A total of 113 DN patients and 100 age- and gender-matched controls were enrolled in the study. The demographic data are presented in Table 3, while the baseline parameters are already reported in our previous article [15].

### 3.2. Sanger Sequencing 

**Figure 2:**
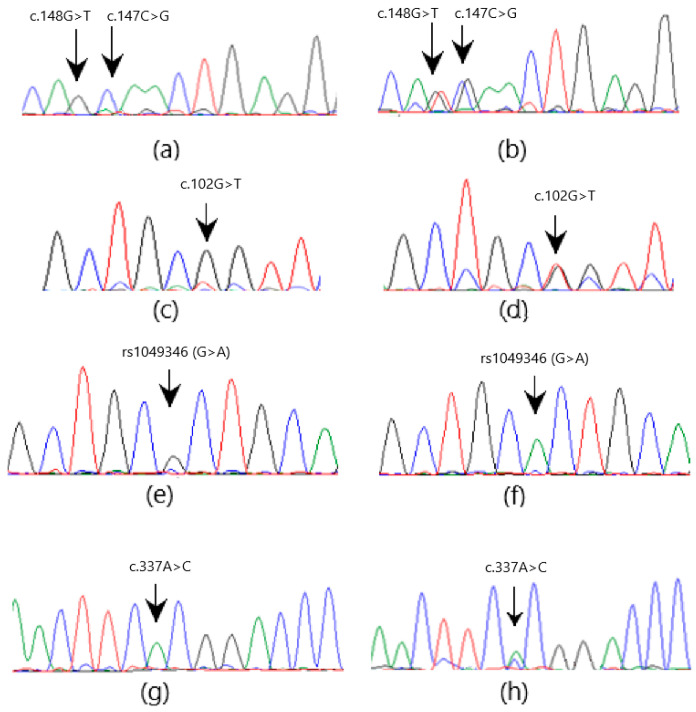
The electrochromatogram shows SNVs in *GLO1* and *ACE* genes. (**a**,**c**,**e**,**g**) are wild-type sequences, while (**b**,**d**,**f**,**h**) show mutated sequences (A = Green, G = Black, T = Red, C = Blue).

**Figure 3:**
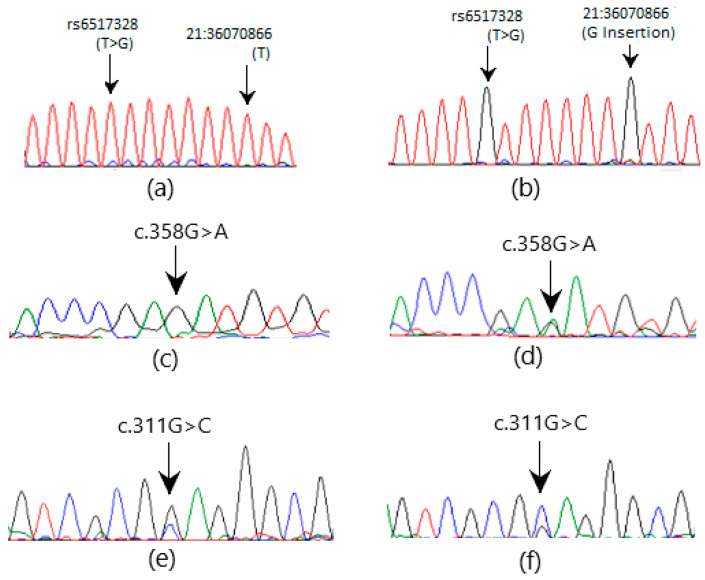
The electrochromatogram shows SNVs in the *CBR1* gene. (**a**,**c**,**e**) are wild-type sequences, while (**b**,**d**,**f**) show mutated sequences (A = Green, G = Black, T = Red, C = Blue).

The Sanger sequencing of *GLO1* exons revealed two heterozygous missense mutations, c.102G>T and c.147C>G, and one heterozygous nonsense mutation c.148G>T in three (2.65%), eight (7.08%), and eight (7.08%) patients, respectively (Figure 2). SNP rs1049346 at location 6:38703061 (G>A) in intron 1-2 of *GLO1* (GRCh38) is another clinically important homozygous SNV observed in 13 (11.5%) patients (Figure 2e,f). In *CBR1*, two heterozygous variations, one missense c.358G>A, and one silent mutation c.311G>C were observed in three (2.65%) and five (4.42%) patients, respectively (Figure 3). A novel change (G insertion) at position 21: 36070866 GRCh38 (NM_001757.4) and rs6517328 (T>G) polymorphisms were observed in intron 1-2 in five (4.42%) and seven (6.19%) patients, respectively (Figure 3). The sequencing of *ACE* exons revealed one heterozygous missense mutation c.337A>C (Figure 2g,h) in three (2.65%) patients. The NM_000789.4 transcript in the human genome database was used as a reference and the NCBI BLAST service was used to check variation. The SNVs observed in the coding and noncoding regions of these genes are presented in Table 4 and Table 5.

### 3.3. Computational Protein–Ligand Docking 

**Figure 4:**
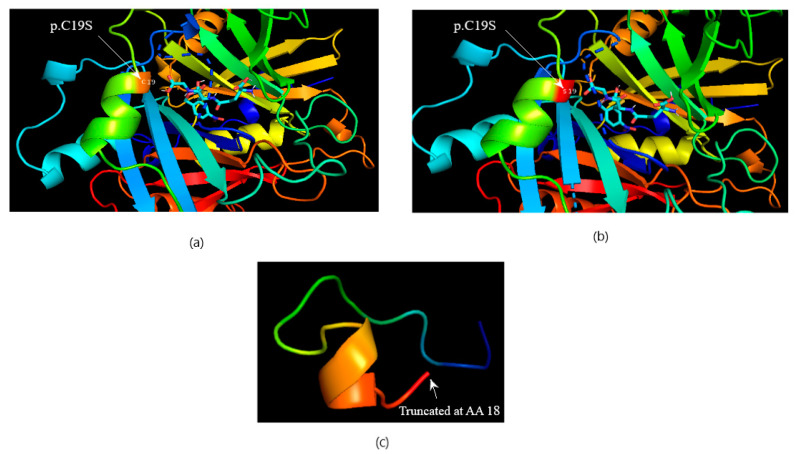
The protein–ligand docking of GLO1 and S-P-Nitrobenzyloxycarbonylglutathione. (**a**) Wild-type protein docking with ligand. (**b**) Mutated C19S. (**c**) Truncated 18AA peptide (stop codon gained).

**Figure 5:**
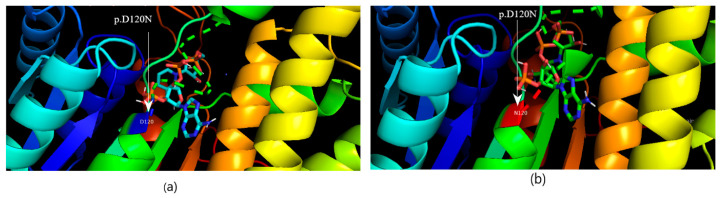
The protein–ligand docking of CBR1 and glutathione with NADPH. (**a**) Normal protein docking with ligand. (**b**) D120N.

**Figure 6:**
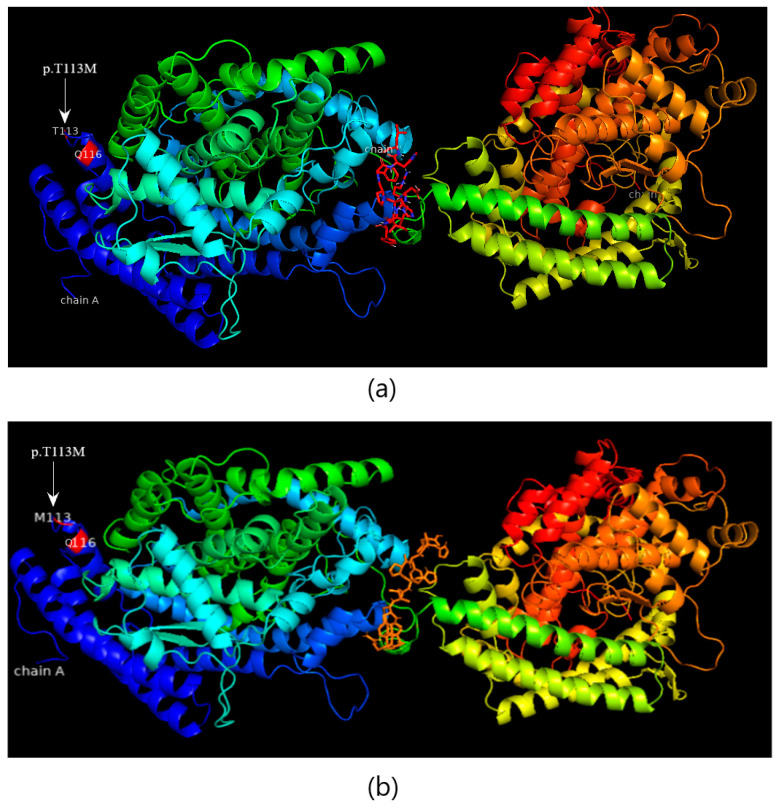
The protein–ligand docking of ACE and angiotensin 1. (**a**) Wild-type protein docking with the ligand. (**b**) T113M novel mutation.

The computational protein model of c.147C>G (C19S) revealed close coordination of this amino acid with the ligand (S-P-Nitrobenzyloxycarbonyl-glutathione) in the protein binding pocket (Figure 4a,b) without an apparent change in protein structure and that of the nonsense mutation revealed truncated protein unable to bind the ligand to the substrate (Figure 4c). Missense variation in the CBR1 enzyme, D120N (c.358G>A), was found to be near the active site/binding pocket of the CBR1 enzyme for ligands (glutathione and NADPH), as shown in the protein in silico structure (Figure 5). Missense variation in ACE T113M (c.337A>C) was not close to its binding site for its ligand (angiotensin 1) and no change in the protein in silico structure was observed in the computational protein model and in silico protein docking with the ligand (Figure 6).

## 4. Discussion

Diabetic nephropathy (DN) remains a critical complication of diabetes mellitus, contributing significantly to morbidity and mortality worldwide. Genetic predisposition plays a pivotal role in the pathogenesis of DN, and investigating the genetic variants and disease susceptibility is imperative.

A novel heterozygous variant c.148G>T (6:38702998) found in *GLO1* was linked with a known heterozygous missense variant at the adjacent position c.147C>G (6:38702999). Both variations were found together in patient samples, and the latter has already been well-reported as a SNP (rs17855424 C>T) at 6:38702999 (GRCh38). However, in this study, a different change (C>G) was found at the same position instead of (C>T), which is again a missense change. In the reported SNV (C>T), the codon changes from UGC to UAC, leading to a change in the amino acid residue cysteine to tyrosine in the glyoxalase-1 protein. However, in this study, the SNV (C>G) led to a change in the codon from UGC to UCC, leading to a change in the amino acid residue from cysteine to serine. This is a novel change and has not been reported before. Cysteine at position 19 changes to serine, which is present near the ligand binding site of the glyoxalase 1 enzyme and may affect the binding, stability, or activity of the enzyme. Moreover, this cysteine residue at position 19 is conserved in different species (when checked using the conservation tool of the UCSC genome browser), indicating its importance for glyoxalase 1 function. Low plasma activity of this protective enzyme has already been reported in patients with severe diabetic nephropathy [15,35,36].

Another missense variation found at 6:38703044 (GRCh38) is a known SNP rs1168871721 (G>A), which causes a change from codon CCG to CUG and a change in amino acids from proline (non-polar residue) to leucine (non-polar residue) in the glyoxalase-1 enzyme. However, in this study, a different novel change from G>T instead of G>A at the same location was observed, which led to the formation of a new codon, CAG, instead of CUG, and the amino acid residue changed to glutamine (polar residue) instead of leucine (non-polar residue) in place of proline (non-polar residue). This SNP is present at the transcription factor-binding site for transcription factors PAX4, HOXB2::PAX1, HOXB2::PAX5, and HOXB2::PAX9 according to NCBI SNP data and the Ensembl genome browser 112 [37]. The amino acid residue proline is conserved at this position in different species (e.g., Rhesus, mouse, dog, elephant) over the evolutionary process, indicating its importance for normal protein structure and function. This change may lead to the ineffective binding of transcription factors or a reduced concentration of the glyoxalase 1 enzyme in circulation, an effect already reported in diabetic nephropathy. This finding is novel, and the related SNP is also not much reported in the literature.

The SNP rs1049346 at location 6:38703061 (GRCh38), found in patients, has already been reported to be linked with decreased levels of circulating glyoxalase-1 and renders carriers susceptible to diseases such as autism [38]. The variant A of the same SNP has been implicated in late-onset epilepsy [39] and acute coronary syndrome (ACS) in diabetic South Indians [40]. However, some newer studies on this subject propose that polymorphisms in the *GLO-1* gene are not linked to low circulating levels of the enzyme or alterations in markers of methylglyoxal-related stress [41].

A novel heterozygous SNV (c. 358 G>A) at position 21:36071018 (GRCh38) in *CBR1* has not been reported in the literature before. This missense variation, D120N, causes codon GAU to change to AAU and the amino acid aspartate (acidic residue) to be replaced by asparagine (polar residue). The computational mutated protein model and in silico ligand interaction show the proximity of this amino acid to the active site and thus may have consequences concerning enzyme activity. Aspartate residue at position 120 is conserved in different species listed in UCSC except in elephants and chickens. The significance of this variation is, however, not listed in ClinVar. A heterozygous change c.311G>C at position 21:36070334 in *CBR1* is a reported SNP (rs25678). Although it is a synonymous variant (L73L), it has been linked with poor enzyme function in acute myeloid leukemia patients [42]. Leucine residue at position 73 is conserved in mammals except elephants and zebrafish. Methyl glyoxal is metabolized by carbonyl reductase 1 as well, and this enzyme’s reduced activity may lead to an accelerated progression of diabetic nephropathy.

It is shown that the *CBR1* gene is helpful in ameliorating the damaging effects of ROS and carbonyl stress and different mechanisms have been demonstrated [12]. The up-regulation of *CBR1* is proposed to reduce lung injury caused by ROS [43] through its protective effects against ROS-triggered carbonyl stress and advanced glycation end products. Newer protein modifiers, e.g., carbonylation, are gaining attention and are aspiring to be enrolled as early markers of DN [44]. This study aimed to elucidate the potential involvement of genetic variants in the *CBR1* gene in patients with early and severe DN.

Two SNVs, 21:36070859 (T>G) and 21:36070866 (G insertion), in *CBR1* were found together in all positive samples and are likely to be independently disease-causing. The former is a reported SNP rs6517328, which is also reported as a cosmic mutation (COSV51737954). These are intron variants and may affect splice site and protein features as per the Ensembl genome browser 112 (available at https://www.ensembl.org/index.html?redirect=no accessed on 20 July 2024). These findings are not reported before and only generally, the *CBR1* gene is implicated in oxidative stress and metabolic syndrome in a few recent studies like [45]. A similar study in mice demonstrated the role of reduced *CBR1* gene expression in mouse liver was causative for diabetes and its effects [46]. The role of carbonyl reductase 1 expression in anti-oxidative mechanisms is proven in vitro as well [47]. On the contrary, a recent study reported that carbonyl reductase-1 enhances glucocorticoid action and thus causes hyperglycemia and is a risk factor for diabetes and hyperglycemia in mice [48]. Polymorphism and genetic variations in this gene have not been studied well for association with diseases, especially diabetic nephropathy. 

Several studies have reported polymorphism in the *ACE* gene, e.g., rs267604983 [18,49] and I/D polymorphism [50], linked with DN. Another study reported that a GG genotype of *ACE* c. 2350 G>A and DD polymorphism of *ACE* I/D were linked with early tubular injury [51]. Though these studies suggest polymorphism in *ACE* is linked to DN, none have demonstrated the novel SNV observed in this study. At position 17:63478018, heterozygous SNV c.337A>C is a novel missense change (T113M) near the N terminal and is far from the ligand binding site and unlikely to affect ligand binding, as revealed by the in silico mutated protein model. This SNV is not reported in the literature. The structure of ACE is preserved in many different species during evolution and its N terminal active site is of prime importance [52]. Changes in protein structure near the N terminal can affect the active site in many ways [52].

## 5. Conclusions

In conclusion, two missense, c.102G>T and c.147C>G, and one nonsense c.148G>T SNVs were found in *GLO1* in patients of early and severe diabetic nephropathy, the latter being most deleterious, resulting in a truncated protein. Missense SNVs c.358G>A in *CBR1* and c.337A>C in *ACE* were observed in patients, leading to potential effects on protein–ligand interaction and enzyme function. The nonsense mutation led to the formation of a truncated protein (GLO1) unable to bind the ligand while two missense mutations altered the protein structure near the active site in CBR1. The affected GLO1 and CBR1 enzymes may lead to the rapid progression of diabetic nephropathy.

### Limitations of the Study

The sample size and sampling technique are not adequate to generalize the findings of this research to the DN population. Only Pakistani subjects were included, so there may be a possibility of ethnic or environmental effects. The identified mutations may be tested in larger cohorts and in other populations to ascertain a clear association of risk.

## Figures and Tables

**Table 1:** Points adhered to while selecting primers online.

Parameter	Target
Tool used	NCBI primer BLAST and Primer 3 plus
Genome	RefSeq representative genome (GRCh38)
Maximum hits in genome	1
5′ self-complementarity	<6
5′ self-complementarity	<6
Single base repetition (max)	3 (no 4 Gs together)
Product size	500 to 900 bp
Minimum length around the target sequence at both ends	150 bp
Primer annealing temperature T_m_	55–63 °C
The maximum difference in T_m_ of the two primers	4 °C
GC content	45 to 60%

**Table 2:** Some primers and their annealing temperatures.

Target	Primer	Primer Sequence	Product Size	Primer T_m_ (°C)	Reaction T_m_ (°C)
*GLO1* Exon 1	Forward	5′ TTCTACCAAATTGCAGCCCTGA 3′	725 bp	61.0	61.8
	Reverse	5′ CAGCCACCGTCGCAACATA 3′		62.3	
*GLO1* Exon 2	Forward	5′ TTGCAAGTTGTAGGTGGTAGGTT 3′	280 bp	60.3	61.2
	Reverse	5′ AAGATGGGTCTGAAAACACTCTC 3′		59.4	
*GLO1* Exon 2 (second set)	Forward	5′ TCTGACACTTTGGACTTGCATCA 3′	761 bp	60.7	61.2
	Reverse	5′ TTTCAGGCTGGCTGGGATAGA 3′		63.6	
*CBR1* Exon 1	Forward	5′ GTCCATAACGCCTCCCTAGG 3′	413 bps	61.3	61.2
	Reverse	5′ GTCCATAACGCCTCCCTAGG 3′		59.3	
*CBR1* Exon 2	Forward	5′ AACTTTGTGTTTCCCTGGCTGGG 3′	812 bp	63.6	64.8
	Reverse	5′ GGATGGACTCCCACGCAGAG 3′		62.3	
*ACE* Exon 1	Forward	5′ AGAGGAGGCCCTTTCTCCAGCT 3′	716 bp	68.6	65.0,
	Reverse	5′ ACCCTCATCCATCCAACTCG 3′		62.9	66.0
*ACE* Exon 2	Forward	5′ TCCGCAAACTAAGGTCTCCC 3′	549 bp	62.7	62.0
	Reverse	5′ TTGGCTTCCTACTCCAGAATGC 3′		61.7	
*ACE* Exon 2 (second set)	Forward	5′ AAGCCCTTGGCCTTCCTC 3′	325 bp	64.2	67.0
	Reverse	5′ CACGATGGGGCACTAGGAG 3′		63.9	

**Table 3:** Demographic factors.

Parameters ^1^		Control Mean (SD) (*n* = 100)	Diabetic Nephropathy Mean (SD) (*n* = 113)	*p* Value
Age	(years)	54.6 ± 10.3	57.2 ± 11.4	0.27
Gender	Male *n* (%)	59 (59)	68 (60.2)	0.13
	Female *n* (%)	41 (41)	45 (39.8)	0.32
BMI		25.5 ± 4.7	27.3 ± 3.2	0.19

^1^ Biochemical parameters are already reported in our previous article [15].

**Table 4:** Genetic variations in coding regions of *GLO1*, *CBR1*, and *ACE* genes (NM_000789.4).

Gene	DNA Sequence Change (GRCh38)	Amino Acid Residue Change	Status	Locus/Site	Type of Change	Control *n* = 100 *n* (%)	DN *n* = 113 *n* (%)
*GLO1*	c.102G>T	p.P4Q	rs1168871721	6:38703044	Missense	0 (0)	3 (2.65)
	c.147C>G	p.C19S	rs17855424	6:38702999	Missense	0 (0)	8 (7.08)
	c.148G>T	p.C19X	Novel	6:38702998	Nonsense	0 (0)	8 (7.08)
*CBR1*	c.358G>A	p.D120N	Novel	21:36071018	Missense	0 (0)	3 (2.65)
	c.311G>C	p.L73L	rs25678	21:36070334	Silent	0 (0)	5 (4.42)
*ACE*	c.337A>C	p.T113M	Novel	17:63478018	Missense	0 (0)	3 (2.65)

**Table 5:** Single nucleotide variations in the noncoding regions of *GLO1*, *CBR1*, and *ACE* genes (NM_000789.4).

Gene	Location (GRCh38)	Variation	Status	Control *n* = 100 *n* (%)	DN *n* = 113 *n* (%)
*GLO1*	6:38703061	G>A	rs1049346	0 (0)	13 (11.5)
	6:38703141	G>T	rs1761734427	0 (0)	1 (0.9)
	6:38703186	C>A	Novel	0 (0)	1 (0.9)
	6:38686709	G>C	Novel	0 (0)	1 (0.9)
	6:38686760	G>A	Novel	0 (0)	1 (0.9)
	6:38686799	G>A	Novel	0 (0)	1 (0.9)
	6:38686823	G>A	Novel	0 (0)	1 (0.9)
	6:38686886	T>C	Novel	0 (0)	1 (0.9)
*CBR1*	21:36070068	G>A	rs11542168	0 (0)	1 (0.9)
	21:36070774	G>A	Novel	0 (0)	1 (0.9)
	21:36070859	T>G	rs6517328	0 (0)	7 (6.19)
	21:36070866	X>G	Novel	0 (0)	5 (4.42)
	21:36070872	X>G	Novel	0 (0)	1 (0.9)
	21:36070881	A>T	rs1469692824	0 (0)	1 (0.9)
	21:36070907	A>C	Novel	0 (0)	1 (0.9)
*ACE*	17:63477094	C>G	rs887305522	0 (0)	1 (0.9)
	17:63477454	C>X	Novel	0 (0)	1 (0.9)
	17:63477926	A>C	Novel	0 (0)	1 (0.9)
	17:63477929	X>C	Novel	0 (0)	2 (1.7)

## Data Availability

The data presented in this study are available upon request from the corresponding authors due to government institutional restrictions.
